# Neurolymphomatosis of the lumbosacral plexus and its branches: case series and literature review

**DOI:** 10.1186/s12885-019-6365-y

**Published:** 2019-11-27

**Authors:** Pierre R. Bourque, Marcos Loreto Sampaio, Jodi Warman-Chardon, Sam Samaan, Carlos Torres

**Affiliations:** 1 Department of Medicine (Neurology), University of Ottawa, Ottawa, Canada; 2 The Ottawa Hospital Research Institute, Ottawa, Canada; 3 Department of Radiology, University of Ottawa, Ottawa, Canada; 4 Division of Nuclear Medicine, The Ottawa Hospital, Ottawa, Canada

**Keywords:** Neurolymphomatosis, Lumbosacral, Neuropathy, B-cell lymphoma, MR neurography

## Abstract

**Background:**

Neurolymphomatosis (NL) is a direct process of invasion of peripheral nerves by lymphoma. It occurs in roughly 5% of patients with lymphoma and represents a particularly difficult diagnostic dilemma when it is the presenting focal manifestation of occult lymphoma.

**Case presentation:**

We present 3 examples of invasion of the lumbosacral plexus and its branches. These cases demonstrate a protean clinical picture with regards to the time relationship to the clinical course of lymphoma and the neuroanatomical extent of lumbosacral plexus invasion. We demonstrate the complementary role of different imaging modalities. A review of the literature summarizes 23 reports where lumbosacral plexus invasion was the index manifestation, at the time of first diagnosis or recurrence of lymphoma. This series confirms the strong preponderance of B-cell type (92%). There is a marked predilection for involvement of the sciatic nerve (74%), either focally or in a longitudinally extensive fashion, from the ischium to the popliteal fossa. There can also be restricted and discrete involvement of tibial and fibular branches. In recent years, ultrasound and CT have been given a more limited role, as screening tools or as a guide for biopsy. MRI neurography and PET-CT have become leading diagnostic modalities for diagnosis, staging and assessment of treatment response.

**Conclusion:**

The diagnosis of NL may be challenging, and it was once only reached at autopsy. Improved diagnostic imaging of focal or even asymptomatic disease offers new hope for earlier diagnosis and successful targeted therapy.

## Background

The term neurolymphomatosis (NL) specifically describes a process of direct endoneurial invasion by lymphoma cells [1–3]. All segments of the peripheral nervous system can be targeted by NL, including cranial nerves, spinal roots, brachial or lumbosacral plexus, and individual peripheral nerve branches [4]. Such focal invasion occurs in roughly 5% of patients with lymphoma, with a strong preponderance of the non-Hodgkin’s B-cell subtype known as diffuse large B-cell lymphoma (DLBCL). NL is a particularly difficult diagnostic dilemma when it is the presenting focal manifestation of occult lymphoma [5].

We present here 3 patients representative of the spectrum of NL of the lumbosacral plexus and its branches. We have surveyed the relevant literature and discuss the role and sensitivity of neuroimaging techniques.

## Case presentations

### Case 1

At age 44, this patient presented for the assessment of painful dysesthesia and progressive asymmetric weakness of upper and lower limbs. He had an unrelated past history of grade 2 astrocytoma treated with cranial radiation and temozolomide, with complete remission.

The following deficits were noted in the right lower limb: grade 4−/5 paresis of plantar flexion, grade 4/5 paresis of hip extension, and absence of the ankle reflex. These deficits were mostly in the distribution of the S1 spinal root, or tibial division of the sciatic nerve. There was additional multifocal paresis and sensory loss in keeping with bilateral brachial plexopathy. Nerve conduction studies showed proximal motor conduction blocks, normal sensory latencies and amplitudes, and patchy EMG findings of acute denervation. These results were interpreted as favoring a multifocal asymmetric demyelinating sensory and motor neuropathy (MADSAM). The diagnosis appeared to be further supported by the finding of bilateral irregular thickening and enhancement of the brachial plexus on MRI. MRI of the lumbar spine and CSF analysis were unrevealing.

**Fig. 1:**
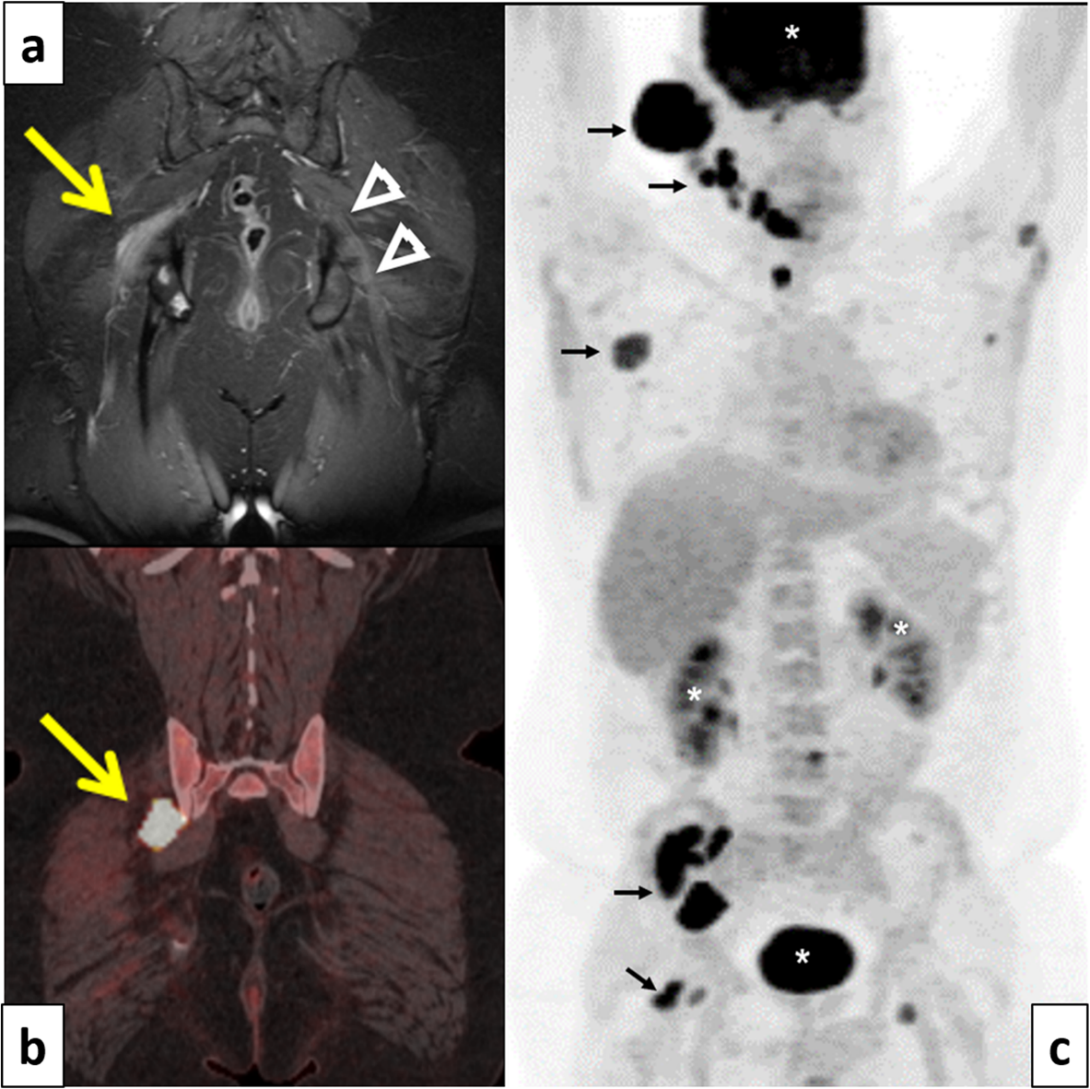
Case 1. 44 years old man. **a** Pelvis coronal contrast-enhanced, fat-suppressed T1- weighted image. Thickening and enhancement of the proximal right sciatic nerve (arrow). Normal contra-lateral side sciatic nerve for comparison (arrow heads). **b** Coronal fused F-18 FDG PET/CT image demonstrating an area of intense hypermetabolic focal activity in the region of the right lumbosacral plexus, in keeping with nodular neurolymphomatosis. **c** Maximum intensity projection (MIP) F-18 FDG PET/CT image demonstrating widespread nodal and extra-nodal hypermetabolic foci (arrows). Note that the intense hypermetabolic activity within the brain, kidneys and urinary bladder are normal findings (*)

There was an initial favorable but only partial response to a combination of intravenous immunoglobulins supplemented by prednisone 60 mg daily. However, eight months after onset, the patient presented with a rapidly expanding external ear mass. Biopsy of this mass led to the diagnosis of diffuse large B-cell lymphoma. The ^18^FDG PET-CT study results and pelvic MRI (Fig. 1) are presented, with emphasis on the lumbosacral involvement.

The patient was treated with conventional chemotherapy, followed by high-dose methotrexate and salvage therapy with gemcitabine and cisplatin. Lower limb weakness improved partially. He also received autologous stem cell bone marrow transplantation. The repeat PET study showed persistent multifocal disease activity and the patient was referred for allogenic bone marrow transplantation.

### Case 2

This 77 years old patient succumbed to complications of peripheral T-cell lymphoma.

He had presented at age 50 with mycosis fungoides. This cutaneous condition was treated over the next two decades with several modalities including topical creams (steroids, nitrogen mustard, acitretin, and imiquimod), electron beam therapy, ultraviolet light therapy and methotrexate. At age 74 he developed a progressive isolated left ulnar neuropathy, initially attributed to entrapment at the level of the elbow. There was no improvement after surgical transposition. At age 75, the patient was reassessed, and fusiform enlargement of the ulnar nerve was demonstrated on ultrasound. Exploration and biopsy were diagnostic of T-cell neurolymphomatosis (CD3 positive lymphocytes within nerve trunks highlighted by the S100 stain). Despite targeted radiotherapy to the left arm, there was further proximal extension of lymphomatosis to the brachial plexus. He was treated with additional involved field radiotherapy and combination gemcitabine/decadron chemotherapy.

**Fig. 2:**
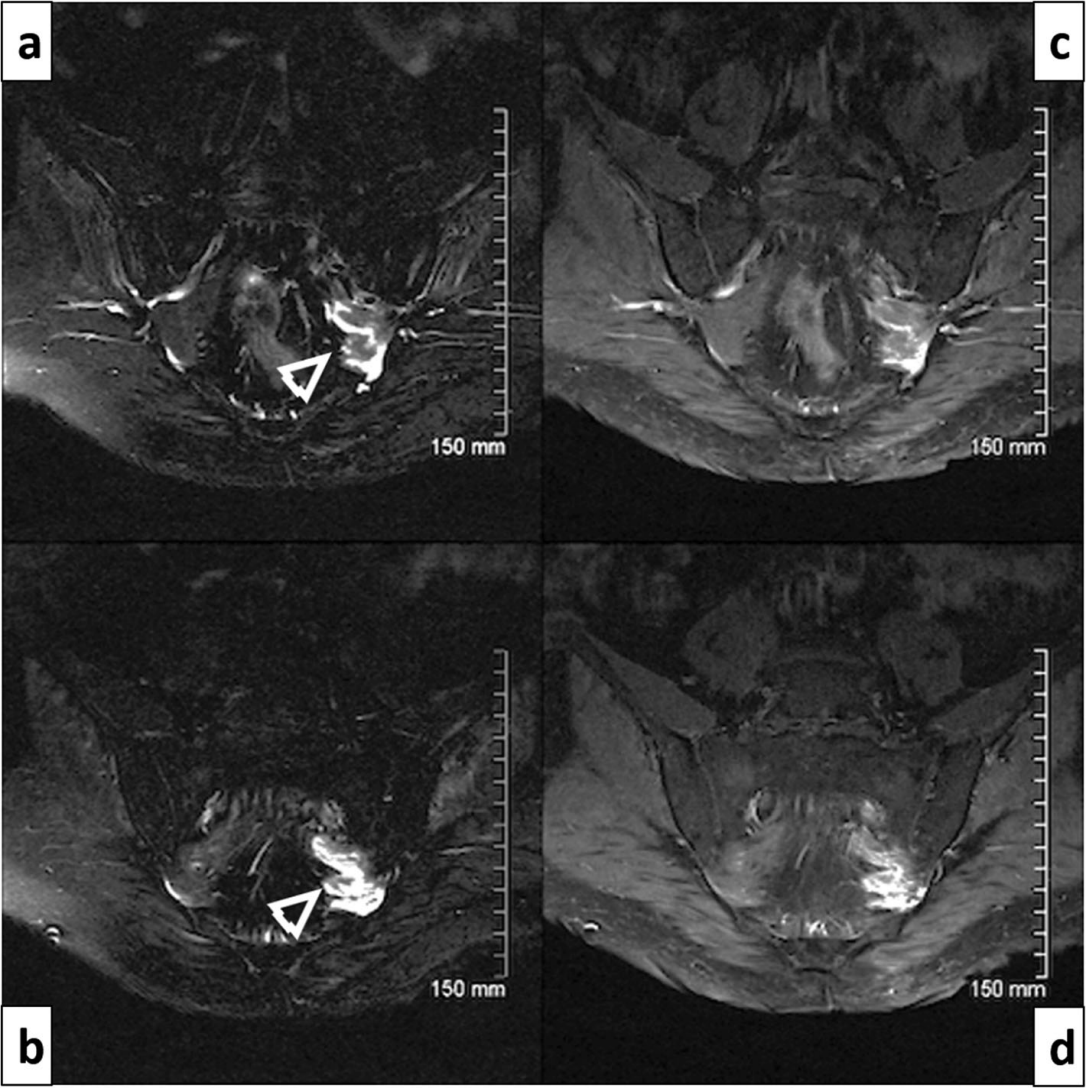
Case 2. 77 years old man. **a** and **b** Coronal oblique STIR images of the lumbosacral plexus showing marked thickening and increased signal intensity of the proximal S1, S2 and S3 nerve roots (arrow heads). **c** and **d** Coronal T1 fat suppressed weighted images post contrast demonstrating enhancement of these sacral roots

At age 76 the patient reported increasingly disabling new neuropathic pain mostly in the posterior aspect of the left lower limb and plantar surface of the foot. His examination showed greater weakness of plantar flexion and eversion (MRC grade 3/5) than dorsiflexion and inversion (4/5). The left ankle reflex was absent. This assessment was in keeping with a left sciatic neuropathy, with greater involvement of the tibial division. MRI of the lumbar spine without contrast revealed only incidental mild changes of spondylarthrosis. A lumbar plexus MRI with gadolinium, however, better characterized thickening, edema and marked enhancement of the left S1-S3 spinal roots (Fig. 2) as well as an infiltrative mass in the region of the left gluteus medius muscle. Palliative radiotherapy only partially controlled his pain, and the patient died a few months later.

### Case 3

This patient was 67 years old when she noticed a palpable baseball-sized lump in the distal posterior right thigh.

She had several significant medical comorbidities including marked obesity, diabetes type 2, hypertension, atrial fibrillation, congestive heart failure and obstructive sleep apnea.

**Fig. 3:**
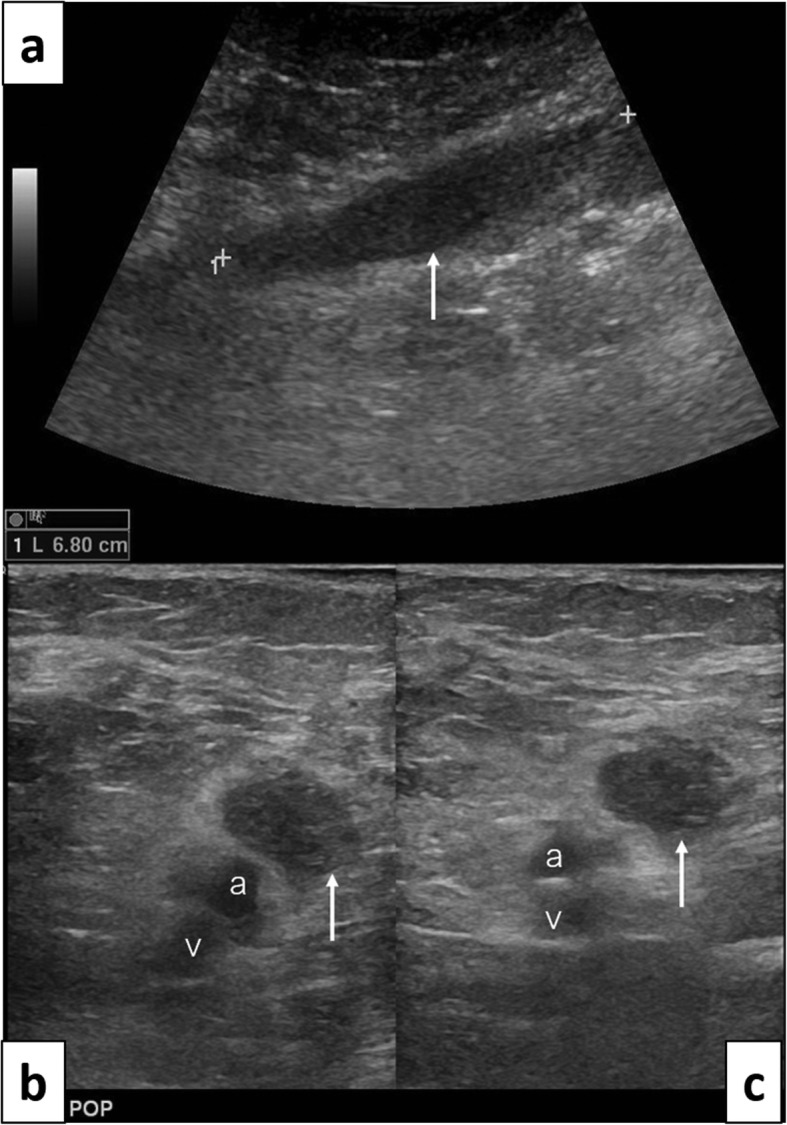
Case 3. 67 years old woman. Ultrasound of the distal thigh/popliteal fossa. **a** Long axis image with convex probe demonstrating a hypoechoic elongated oval-shaped longitudinally oriented mass (arrow). This corresponded to the course of the sciatic nerve. **b** and **c** Short axis view of the same lesion with high resolution 12 MHz linear probe without and with probe compression, confirming the presence of a non-compressible hypoechoic lesion (arrows), adjacent to the popliteal artery (a) and vein (v). Note the partial collapse of the popliteal vein (v) in (**c**)

**Fig. 4:**
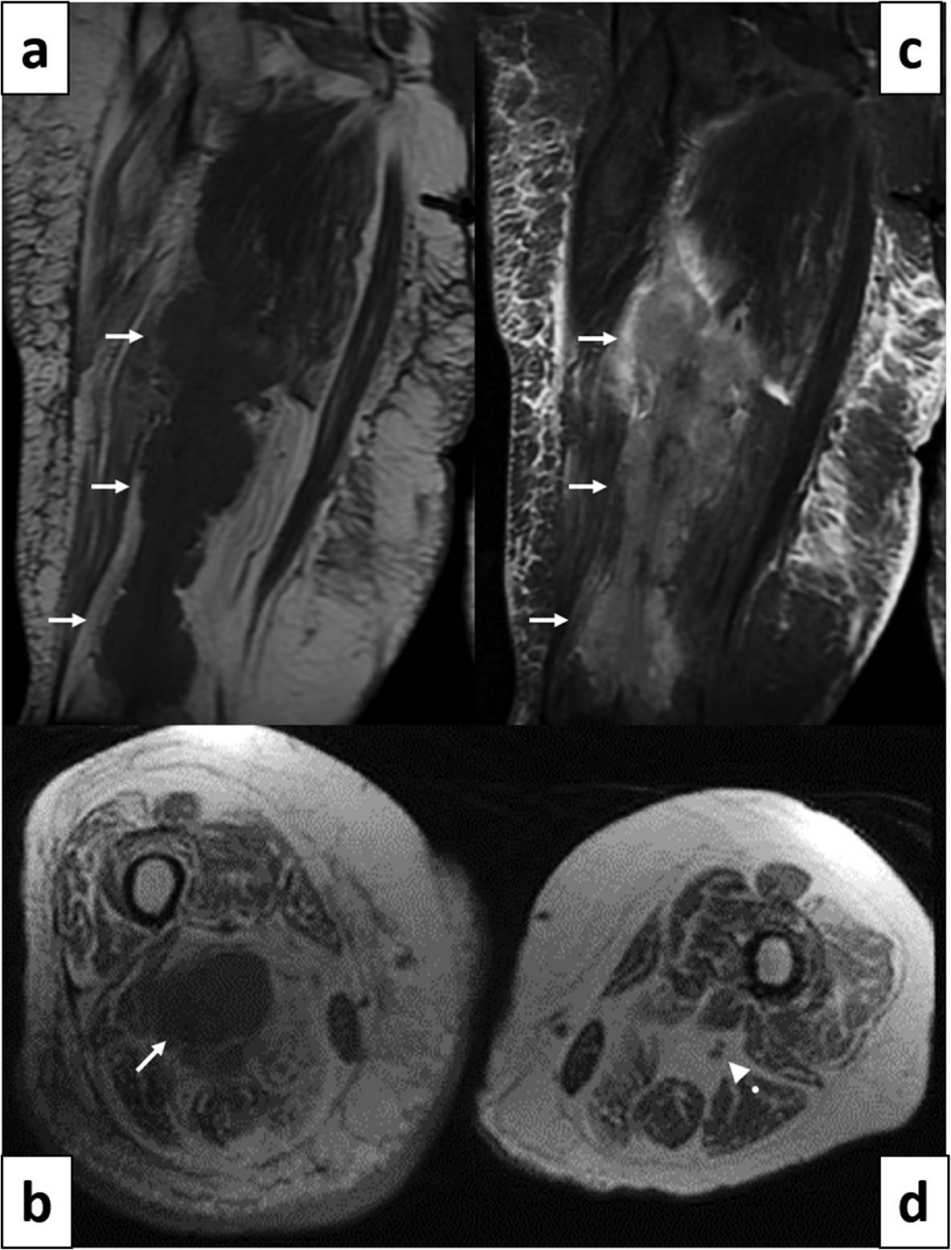
Case 3. 67 years old woman. Diffuse multi-lobular mass along the right sciatic nerve on MRI. **a** Coronal T1-weighted imaging demonstrating irregular hypointense thickening of the right sciatic nerve (arrows) and diffuse fatty infiltration of the same muscles. **b** Coronal STIR imaging demonstrating corresponding thickening and increased signal intensity of the sciatic nerve (arrows). **c** Axial T1-weighted imaging showing thickening of the right sciatic nerve (arrow). The normal contra-lateral sciatic nerve is also observed (arrow head). Please note the marked asymmetry in the diameter of the thighs, right larger than left

**Fig. 5:**
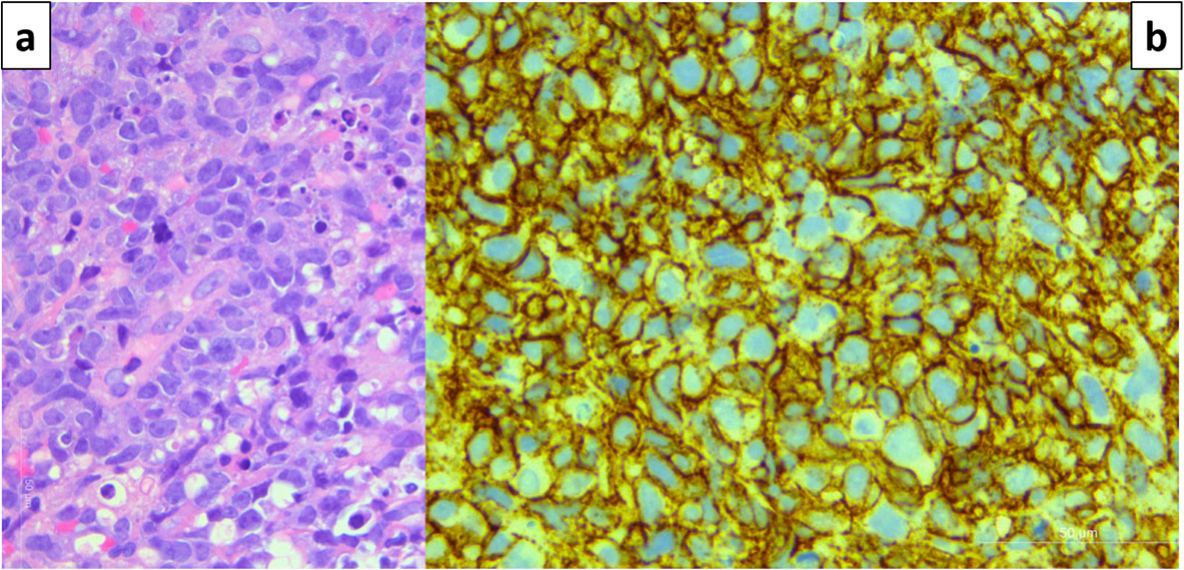
Case 3. 67 years old woman. Ultrasound guided biopsy of the thigh mass. **a** H&E. The tumor is composed of a diffuse infiltrate of large lymphoid cells with irregular nuclei, prominent nucleoli and numerous apoptotic bodies (arrow). **b** The tumor cells strongly express membranous CD20, a pan B-cell marker, shown here. They also expressed bcl-2, MUM-1/IRF4, bcl-6. Labelling with Ki-67 was > 90%

When referred for neurosurgical evaluation, she was found to have complete paralysis of all right ankle movement, but preserved hip and knee flexors and extensors. There was sensory loss in the sciatic territory, with sparing of the femoral cutaneous innervation. The right ankle reflex was absent. Ultrasound (US) of the popliteal fossa (Fig. 3) followed by an MRI of the thigh (Fig. 4) showed longitudinally extensive lobular enlargement of the sciatic nerve from the ischium to the popliteal fossa (measured at 5.1 × 7.5 × 28 cm). The patient underwent urgent ultrasound-guided biopsy, which was in keeping with a diffuse large B-cell lymphoma (Fig. 5).

**Fig. 6:**
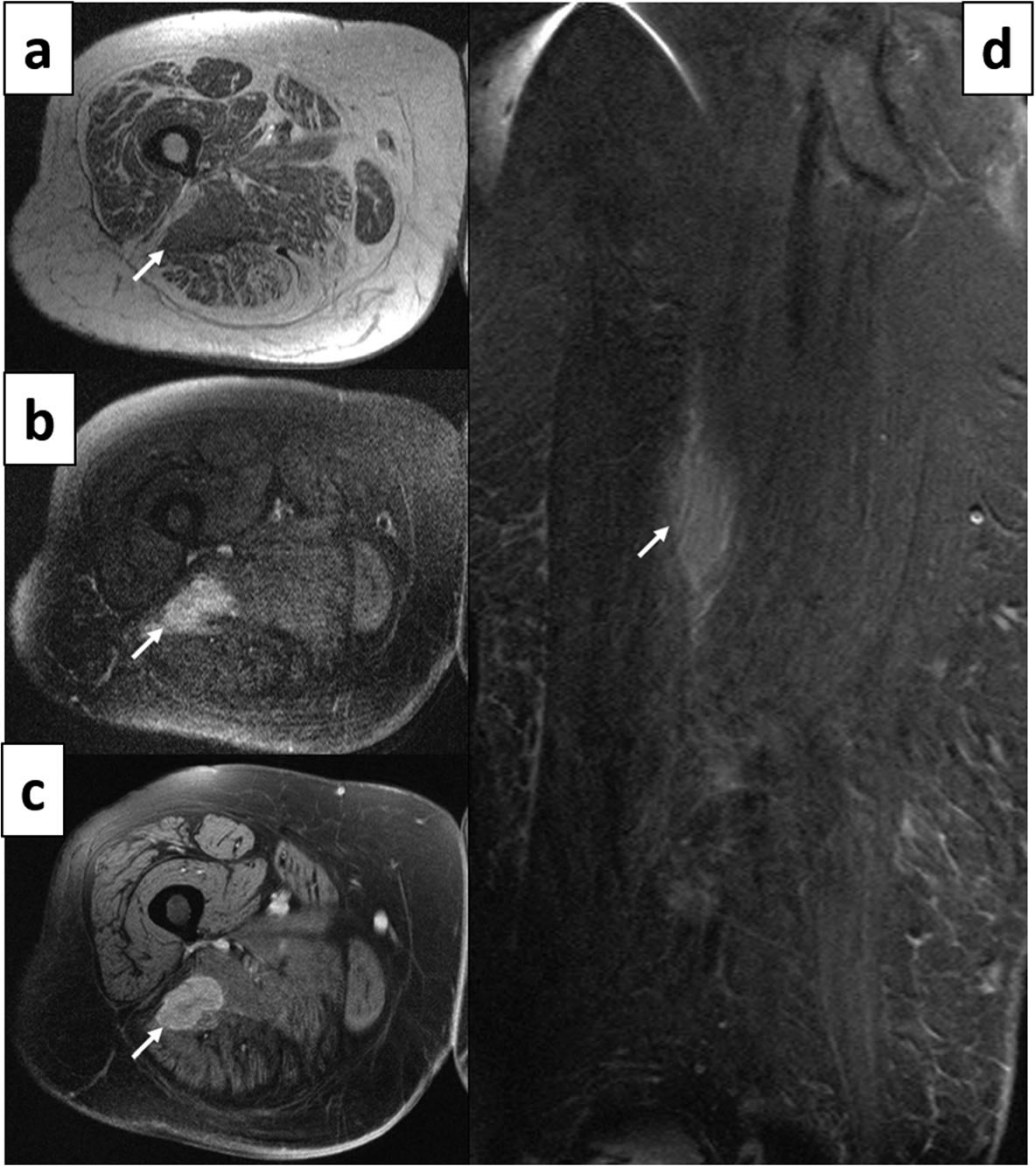
Case 3. 67 years old woman. 11 months post treatment follow-up MRI of the sciatic mass. **a** The axial T1 weighted image shows the hypointense mass within the right sciatic nerve (arrow) in the mid-thigh region. **b** The mass shows increased signal intensity in the fat-suppressed T2 weighted image and **c** post gadolinium enhancement in the fat- suppressed T1-weighted image (arrows in **b** and **c**). **d** Coronal STIR imaging demonstrating good response to treatment, with significant interval decrease in size of the tumor (arrow)

Treatment was initiated with five cycles of rituximab, cyclophosphamide, doxorubicin, vincristine and prednisolone. There was no clinical improvement with regards to deficits of sciatic neuropathy, but repeat MR imaging (Fig. 6) showed marked reduction in tumor size, now measured at 2 × 3.7 × 6.4 cm. The patient could not follow the follow-up management plan of the hematology consultant, because of intercurrent medical complications, including a surgery for bowel obstruction from an incarcerated hernia. When reassessed at age 68, a new MRI showed marked re-expansion of the thigh mass, with dimensions of 15.5 cm × 12.5 cm × 18.5 cm. A palliative treatment plan was recommended, and the patient was referred back to her community hospital.

## Discussion and conclusions

In advanced biopsy-proven recurrent or treatment-resistant diffuse large B-cell lymphoma, the occurrence of multifocal radiculopathy, plexopathy or mononeuropathies will immediately suggest a diagnosis of NL [6]. In contrast, the diagnosis may be elusive in the setting of isolated focal progressive peripheral neuropathy if the patient has no history of malignancy or has achieved prolonged lymphoma remission after therapy. Our series is representative of this clinical spectrum of NL presentation. In case 3, lumbosacral NL was the first symptomatic focus of lymphoma, while in cases 1 and 2, lumbosacral NL was a secondary finding in disseminated or upper limb predominant disease. We also present the use of multiple imaging modalities for diagnosis and work-up of the patient during different stages and severity of the disease. On the other hand, we do not present an extremely granular timeline of all events for each patient given the redundancy with the provided clinical information and also the complexity of the cases.

In the absence of documented systemic malignancy, the differential diagnosis of progressive focal neuropathy will include a long list of benign or malignant peripheral nerve sheath tumors [7], perineurioma [8], entrapment syndromes, multifocal syndromes of inflammatory demyelinating neuropathy [9], nerve vasculitis and sarcoidosis [10]. Lymphomatous infiltration should however always be also considered.

In Table 1, we have summarized 23 case reports where isolated lumbosacral plexus NL occurred de novo or as the index manifestation of recurrent lymphoma. As expected, B-cell histopathology was reported in 21 cases (92.3%), and T-cell lymphoma was noted in only 2 cases [19, 28]. The median age at presentation was 62 years, compared to 70 years in a large UK epidemiological survey for diffuse large B-cell lymphoma [32]. Primary sciatic localization was by far most frequent (17, or 73.9%), followed by posterior tibial nerve (8.6%) [11, 12], common or deep fibular nerve (8.6%) [13, 17] and the pelvic segment of the femoral nerve (4.3%) [15]. Isolated lumbosacral plexus branch NL infiltration was the first clinical manifestation of lymphoma in 17 of 23 cases, and was reported in the setting of recurrent lymphoma in six cases. Sciatic involvement presented as a discrete or lobulated nerve infiltration at any level of the thigh. In three cases, infiltration of the sciatic nerve was diffuse at time of diagnosis, extending from the ischial region to the popliteal fossa, and then further caudally into the tibial nerve [26, 27, 29]. In contrast, the most discrete examples of lower limb NL were cases of isolated short-segment infiltration of the tibial nerve at the ankle [11] and deep fibular nerve in the mid lower leg [13]. Our table does not list treatment outcomes, as most reports only documented the initial favorable response to radiotherapy or chemotherapy without providing sufficient longitudinal data. Death from disseminated disease within a few months to 3 years was however specifically mentioned in 5 cases in this series and occurred in two of our cases.

**Table 1:** Chronological summary of 23 cases where lumbosacral neurolymphomatosis was the leading diagnostic feature of lymphoma, or an index manifestation at time of lymphoma recurrence

Reference First author, year	Age sex	MRI features ^a^	Cell type	Anatomical location	Relation to diagnosis
(*blinded information*), 2018 (present article)	67 F	Iso T1, ↑T2, homogeneous G+, PET+	B	R Sciatic (entire thigh)	P^b^
Moussa, 2018 [11]	80 F	↓ T1,Int -↑ T2 PET focal +	B	Posterior tibial (ankle) 3.7 × 3.1 × 3.8 cm	P
Lee, 2016 [12]	77 M	↓ T1, Int T2, Subtle periph G+, PET+	B	L Post tibial (knee) 7.2 × 6.7 × 2 cm	P
Sideras, 2016 [13]	65 F	Int T1, ↑ T2, Mild G+, PET+	B	R Deep peroneal (mid leg) [13]4.9 × 1.2 × 1.0 cm	P
Shree, 2016 [14]	68 M	Strong G+ PET +	B	R sciatic (mid thigh)	R^c^
Saito, 2014 [15]	61 F	PET +	B	R Femoral (pelvis)	R
Deivaraju, 2014 [16]	23 F	↑ T2	B	L Sciatic (mid thigh)	R
Koyama, 2010 [17]	74 F	↑ T1, PET+	B	R Common peron (knee)	R
Kahraman, 2010 [18]	63 F	↑ T2, G+ (diffuse pattern)	B	L Sciatic (proximal)	P
Kosa, 2009 [19]	60 M	NS	T	Bilateral Sciatic (distal, discrete)	R
Strobel, 2007 [20]	59 -	NS	B	L sciatic (entire thigh)	P
Rota, 2006 [21]	44 F	↑T2, G+	B	R sciatic (proximal thigh)	P
Descamps, 2006 [22]	55 M	↑T2	B	L sciatic (entire thigh)	P
Preston, 2001 [23]	52 M	↑T2, G +	B	L sciatic (mid thigh)	P
Moore, 2001 [24]	69 M	↑T2, G +	B	R lumbosacral radiculo plexopathy	R
Misdraji, 2000 [25]	62 F	↓T1, ↓↑ T2	B	L Sciatic (NS)	P
Misdraji, 2000 [25]	49 M	NS	B	R Sciatic (NS)	P
Quinones, 2000 [26]	52 M	↓T1, ↑T2, Slight G+	B	R Sciatic (entire thigh and proximal tibial branch)	P
Roncaroli, 1997 [27]	44 M	NS	B	L Sciatic (lower thigh), 5 cm segment	P
Masahiko, 1995 [28]	34 M	↓T1, marked G+.	T	L Sciatic (entire thigh)	P
Eusebi, 1990 [29]	72 M	NS	B	Sciatic (Ischium to lower tibial nerve)	P
Pillay, 1988 [30]	61 M	↑T1, ↑T2	B	L Sciatic (proximal thigh) 4 cm segment	P
Purohit, 1986 [31]	64 F	NS	B	R Sciatic (lower half) 10 cm segment	P

^a^ MRI signal intensity (in relation to neighboring muscles: ↑ = increased; ↓ = decreased; Int = Intermediate; iso = isointense; G+ = Gadolinium enhancement); PET+ = Positive Positron Emission Tomography

^b^P = Presenting manifestation of lymphoma

^c^R = index manifestation at time of Recurrent disease

In the imaging assessment of nerve tumors and the detection of NL, ultrasound is still valuable because of its easy access, safety, lack of contraindications and favorable patient tolerability [14]. It can reliably distinguish cystic from solid masses, the anatomical localization including the relationship to blood vessels and help guide safe biopsy. Additionally, color Doppler ultrasound has been proposed to demonstrate increased blood flow in NL, but less in entrapment or inflammatory neuropathy [16, 18]. Enhanced CT imaging has been largely superseded by MRI, when the latter is not contraindicated. CT may however more easily detect tumor calcifications and may also help characterize tumors in relation to neighboring bony structures, particularly at the level of the spine and thorax [20]. CT has also been the modality most commonly combined with PET to improve spatial resolution.

MRI neurography is emerging as a powerful tool to help detect and characterize nerve pathology [21]. Normal nerves show gradual tapering, remaining typically smaller than the accompanying artery. They are usually outlined by fat (best appreciated on T1-weighted images) and are isointense to skeletal muscle on both T1 and T2-weighted images. They have an even fascicular distribution, with no appreciable gadolinium enhancement other than at the level of dorsal root ganglia. The more prevalent forms of diffuse metabolic or toxic axonal polyneuropathy do not show significant deviation from this normal pattern. Many focal inflammatory, infiltrative, hypertrophic and neoplastic neuropathies however show a fairly consistent pattern of MRI abnormality [22, 23]. Such disorders often feature focal or diffuse nerve enlargement, often exceeding the accompanying artery. In these disorders, abnormal nerves remain isointense to muscle on T1-weighted images, but are often significantly hyperintense on T2-weighted or STIR images. They may also show marked fascicular disorganization, deviation from their normal course and significant enhancement. MRI changes of denervation of neighboring muscles may provide an additional clue to the presence of neuropathy. These criteria however offer relatively poor specificity to help discriminate between NL and other etiologies such as acute or chronic inflammatory demyelination, inherited hypertrophic neuropathy, radiation changes, perineuroma, focal hypertrophic neuritis, amyloidosis or neurofibromatosis. As a general rule, gadolinium enhancement is absent or minimal in amyloid or genetic neuropathy. It tends to be modest in acute inflammation or radiation, but it may be quite prominent in chronic inflammatory demyelinating polyneuropathy (CIDP), infectious neuropathy, perineuroma and NL [23]. Within the limits of resolution of MRI, it may be challenging to distinguish an extraneural soft tissue lymphomatous infiltration from true intraneural spread. The demonstration of thickening and enhancement of distal peripheral nerve branches or proximal radiculo-plexus elements may strongly suggest NL [2].

Baehring reported an MRI diagnostic sensitivity of 70% in a series of 40 cases of NL, combining the experience at the Massachusetts General Hospital and a review of the literature up to the year 2000 [1]. Most modern MR protocols routinely take advantage of more comprehensive image protocols (T1, T2, fat saturation, STIR), reduced slice thickness (1–3 mm), 1.5 T- 3 T magnet strength and multi-planar capability. In a review covering the period of 2001–2008, Grisariu reported a slightly higher sensitivity of 80%, likely reflecting such technical improvements in image acquisition [2].

^18^FDG-PET/CT is a very sensitive technique to reveal hypermetabolic foci, with one study reporting detection of at least one active site in 97% of patients with diffuse large B-cell lymphoma [24]. ^18^FDG-PET/CT has also been shown to be superior to MRI to detect bone marrow involvement in lymphoma [25]. PET imaging routinely provides a whole-body field of imaging and is uniquely suited to detect both nodal and extranodal tumor invasion. In a literature case review covering the 2001–2008-time period, NL was detected by PET in 90% of cases. In our review of lumbosacral NL (Table), PET was positive in 7/7 reports where it was done. Note that all such reports were from the current decade, reflecting the now recent use of PET in lymphoma screening. ^18^FDG-PET by itself is a highly sensitive screening tool which offers poor focal spatial resolution, thus it is commonly correlated with CT. Targeted MRI neurography at sites of hypermetabolism can be expected to substantially further improve the anatomical characterization of intraneural invasion. In addition, ^18^FDG-PET has been demonstrated to be helpful in monitoring the response to therapy [30, 31].

Neurolymphomatosis was once a diagnosis often only reached at autopsy. Advances in neuroimaging offer hope for early recognition, at a stage of discrete unifocal or even entirely asymptomatic lymphoma invasion. Survival rates remain poor with recurrent disease but newer protocols offer hope by combining high-intensity chemotherapy, targeted radiotherapy, immunotherapy (such as chimeric antigen receptor T-Cell) and salvage bone marrow transplantation [33].

## Data Availability

Not applicable.

## Abbreviations

DLBCL: diffuse large B-cell lymphoma
MADSAM: multifocal asymmetric demyelinating sensory and motor neuropathy
MIP: maximum intensity projection
NL: neurolymphomatosis
US: ultrasound
